# Dynamic mechanisms and targeted interventions in cerebral ischemia–reperfusion injury: pathological cascade from ischemia to reperfusion and promising therapeutic strategies

**DOI:** 10.3389/fnins.2025.1649533

**Published:** 2025-08-18

**Authors:** Xufeng Meng, Zhi Zheng, Li Yang, Chen Yang, Xiaoli Li, Yunfei Hao

**Affiliations:** ^1^First Clinical Medicine College, College of Integrated Traditional Chinese and Western Medicine, Gansu University of Traditional Chinese Medicine, Lanzhou, China; ^2^Key Laboratory of Cerebrovascular Diseases of Gansu Province, Cerebrovascular Disease Center, Gansu Provincial People’s Hospital, Lanzhou, China; ^3^Lanzhou University, Lanzhou, China

**Keywords:** cerebral ischemia reperfusion injury (CI/RI), ischemic stroke (IS), pathologic process, mitochondrial dysfunction, inflammation

## Abstract

Cerebral ischemia–reperfusion injury (CI/RI) is a critical event causing secondary neurological deterioration following vascular recanalization in patients with ischemic stroke (IS), involving multiple interrelated pathological processes that synergistically aggravate brain injury. However, the underlying mechanisms remain incompletely elucidated, necessitating systematic investigation. This review systematically elucidates the dynamic pathological mechanisms underlying CI/RI during ischemic and reperfusion phases. Hypoxia-induced mitochondrial energy failure and TLR4/NF-κB-mediated inflammation predominate in the ischemic phase, while reperfusion triggers a reactive oxygen species (ROS) burst, amplifying oxidative stress (OS). These interconnected cascades form a self-perpetuating pathological loop. Targeting these pathways, therapies such as the TLR4 antagonist ApTOLL, the iron chelator deferoxamine, and the free radical scavenger Edaravone have shown promise. Nevertheless, significant challenges persist, including single-target limitations, poor delivery efficiency across the blood–brain barrier, and insufficient mechanistic insights. By integrating dynamic mechanisms and corresponding therapeutic strategies, this review summarizes recent advances in understanding the core pathological mechanisms and targeted interventions for CI/RI, discusses the current status and future prospects of these mechanisms and therapies, and aims to provide a systematic framework for mechanistic insights into CI/RI and a theoretical foundation for its precision treatment.

## 1 Introduction

Intracranial vascular thrombosis, resulting from endothelial dysfunction or hemodynamic abnormalities, leads to cerebral hypoperfusion, causing a critical lack of oxygen and glucose in brain tissue. This subsequently results in neurological deficits in corresponding areas, culminating in ischemic stroke (IS). This condition is characterized by abrupt onset, high mortality, and disability. In 2019 globally, stroke remained the second-leading cause of death and the third-leading cause of disability (Feigin et al., 2021). Among all stroke subtypes, IS accounts for 87% of cases (Tsao et al., 2023). For ischemic stroke, timely restoration of blood flow for reperfusion is the key to treatment. To date, methods such as intravenous thrombolysis and mechanical thrombectomy have been proven to promote vascular recanalization and significantly improve clinical outcomes (Herpich and Rincon, 2020; Yuan et al., 2021). While these interventions are effective, growing evidence suggests that reperfusion of blood flow at the site of ischemia can lead to secondary damage, and the recovery of blood flow and oxygen supply is often associated with exacerbating tissue damage, a process known as cerebral ischemia–reperfusion injury (CI/RI) (Gauberti et al., 2018; He et al., 2020).

**Figure 1:**
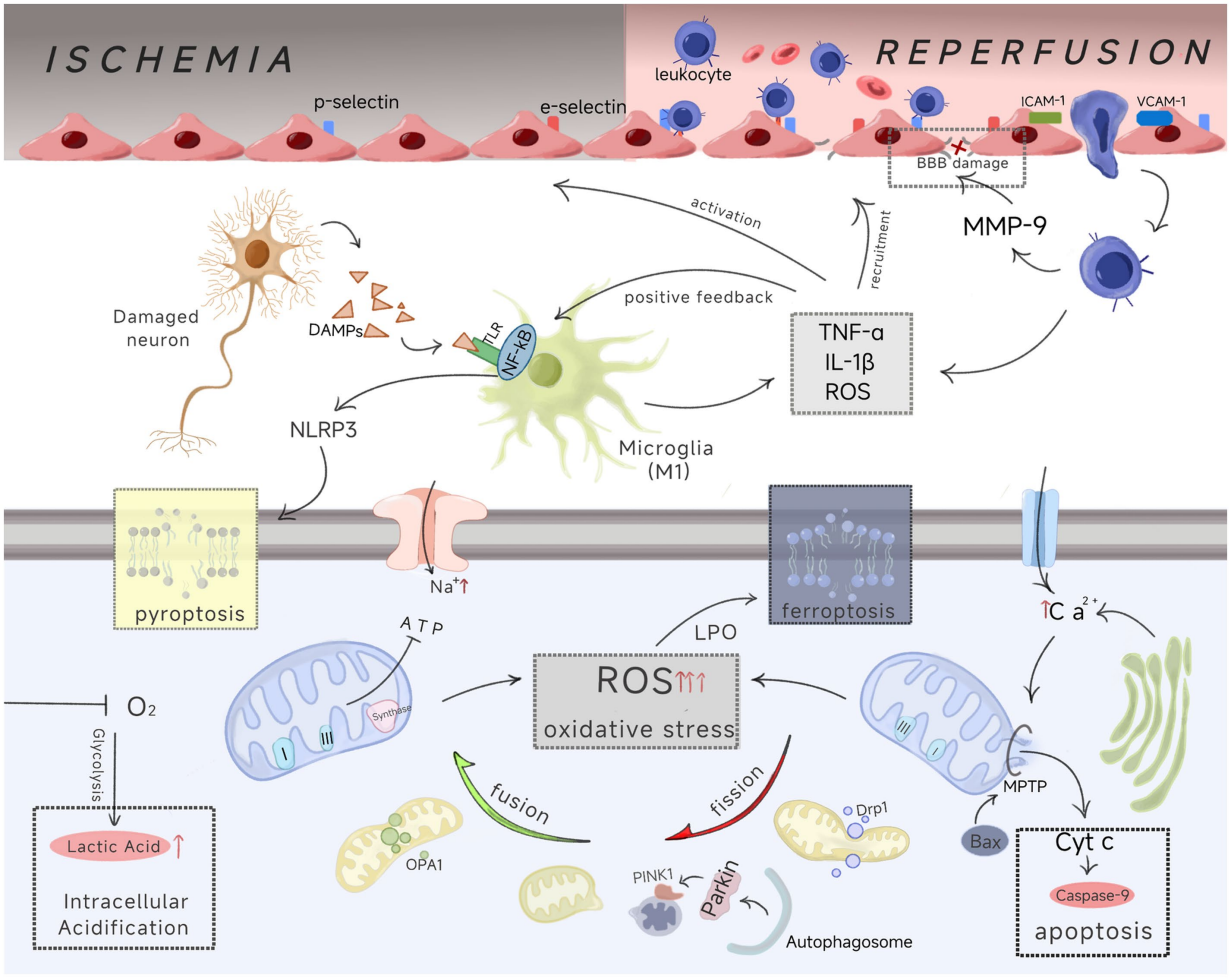
The figure visually depicts the initiation and progression of inflammatory responses, oxidative stress, and mitochondrial dysfunction in CI/RI and demonstrates the interplay between various pathological processes. The upper part of the figure shows blood flow status, the middle part shows the inflammatory cascade, and the lower part shows a series of pathological activities occurring within cells.

The mechanism of cerebral ischemia–reperfusion injury is complex, involving multiple pathological processes, such as inflammatory response, oxidative stress, mitochondrial dysfunction, blood–brain barrier (BBB) damage, vascular regeneration, and programmed cell death, such as apoptosis, pyroptosis, and ferroptosis (Huang et al., 2022; Zhang et al., 2024a). These pathological processes run through the entire process of brain tissue from ischemia to reperfusion, ultimately causing irreversible damage to brain tissue. And there is a mutual influence and interweaving relationship between them, such as mitochondrial damage promoting the production of ROS and exacerbating oxidative stress, which in turn can activate inflammatory reactions, and persistent inflammatory damage will injure the blood–brain barrier (Cao et al., 2023; Zorov et al., 2006). Furthermore, cytochrome C released during the opening of mitochondrial permeability transition pores (MPTP) can activate ATP-dependent caspase to induce neuronal apoptosis (Kim et al., 2003) (Figure 1). The interconnection and interplay of multiple pathological processes have greatly increased the difficulty of treating CI/RI. In the current context of precision medicine, more and more studies are focusing on pathological cascades and molecular transduction mechanisms in CI/RI, by targeting upstream signaling pathways such as phosphatidylinositol 3-kinase/protein kinase (PI3K/Akt) (Tao et al., 2025; Zhou et al., 2024a), nuclear factor kappa B (NF-kB) (Gong et al., 2023; Li et al., 2023d), Janus kinase/activator of signal transduction and activation of transcription (JAK/STAT) (Dong et al., 2021), mitogen-activated protein kinase (MAPK) (Lu et al., 2018; Wang et al., 2020; Zhou et al., 2020), Notch (Shi et al., 2017; Wang et al., 2019; Zhang et al., 2014), as well as key pathological processes, new therapeutic strategies for mitigating CI/RI are provided while revealing the molecular mechanisms of injury. This article summarizes and sorts out the main pathological processes in CI/RI, cites relevant research evidence, and aims to elucidate their dynamic mechanisms in CI/RI.

In summary, due to the complexity and diversity of injuries during the CI/RI process, as well as the interweaving of the temporal and spatial occurrence of each pathological stage, effective interventions targeting the pathological cascade are crucial for the regulation of injuries.

## 2 The complex pathological processes in CI/RI

### 2.1 Inflammation

Inflammation response is an important pathological process that promotes the onset and progression of IC/RI, leading to brain tissue injury (Zhang et al., 2025), and neuroinflammation is involved in the entire pathophysiological process of this disease (Liu et al., 2021). The brain belongs to a sterile organ, and inflammation in cerebral ischemia–reperfusion injury is aseptic inflammation, which is unlike inflammation caused by pathogen infection, and is caused by the activation of infiltrating myeloid cells due to necrotic brain cells, leading to aseptic inflammation (Sakai and Shichita, 2023).

#### 2.1.1 Ischemic stage

After the onset of cerebral ischemia, dead cells and macrophages actively secrete DAMPs (damage-associated molecular patterns) (Gong et al., 2020), which include high mobility group box 1 (HMGB1), s100, and ATP, and play a role in initiating immune responses after central nervous system injury (An et al., 2014). It has been shown that the leukocyte accumulation correlates with the amount of HMGB1 and s100 (Schuhmann et al., 2021). Microglia make up 5–20% of the total glial cell population and act as resident immune cells in the brain (Jayaraj et al., 2019), which are rapidly activated within minutes of cerebral ischemia–reperfusion (Cao et al., 2021). They are the first responders to neuroinflammation and can change their phenotype according to different environments (Madore et al., 2020). Microglia can recognize pathogen-associated molecular patterns (PAMPs) and DAMPs by expressing toll-like receptors (TLRs), nucleotide binding oligomerization domain (NOD) like receptors (NLRs), and retinoic acid-induced gene-1 (RIG-1) like receptors (rlr) (Jurcau and Simion, 2021). TLR activation can stimulate the recruitment of a cell specific adaptor protein, the Myeloid Differentiation Factor 88 (MyD88), which mainly induces the nuclear factor kappa B (NF-κB) signaling pathway, whereas rlr activates mitochondrial antiviral signalling proteins (MAVS) and tumor necrosis factor (TNF) receptor-associated factors (TRAFs) to trigger the NF-kB signalling pathway (ElAli and Rivest, 2016). Activation of these downstream pathways drives a cascade of inflammatory responses. Meanwhile, activated microglia can be converted into M1 (pro-inflammatory type), releasing a variety of deleterious substances such as interleukin-1β (IL-1β), TNF-*α*, and reactive oxygen species (ROS) into the circulation (Perego et al., 2011), and these pro-inflammatory factors can recruit leukocytes to the site of injury, exacerbating brain damage and leading to cell death (Figure 1). In addition, endothelial cells can be activated by IL-1β and TNF-α molecules, and activated endothelial cells can express and expose adhesion molecules, including p-selectin, e-selectin, and L-selectin within a few hours (Przykaza, 2021), which is more conducive to the rolling and adhesion of white blood cells. Moreover, the tight junctions between the BBB, which protects the brain and maintains the stability of the brain’s internal environment, and the endothelial cells become permeable after brain injury, creating favorable conditions for peripheral immune cells to infiltrate the brain parenchyma (Tobin et al., 2014). These processes set the foundation for the infiltration of activated inflammatory cells and further amplification of inflammatory responses during reperfusion.

#### 2.1.2 Rperfusion stage

After reperfusion, ROS increased further, and the recruited leukocytes crossed the BBB into the brain parenchyma via intercellular adhesion molecule 1 (ICAM-1) and vascular cell adhesion molecule 1 (VCAM-1) overexpressed on the surface of the endothelial cells (Tirandi et al., 2023). These cells can continue to release pro-inflammatory mediators, ROS, Metalloproteinases (especially MMP-9), leading to increased brain edema and damage to the blood–brain barrier (Pluta et al., 2021) (Figure 1). During the reperfusion phase, microglia and recruited macrophages can phagocytose and remove cell debris and dying cells, but living cells in the ischemic penumbra under sublethal conditions can also be phagocytosed and die due to exposure to the “eat me” signal phosphatidylserine (PS) (Neher et al., 2013). At the same time, an increase in pro-inflammatory cytokines will activate the NF-kB pathway through a positive feedback loop, causing cytokine storms and exacerbating brain tissue damage.

In conclusion, the pathological cascade of the inflammatory response is closely related to the increase of pro-inflammatory factors and adhesion molecules, which provide the environment and conditions for inflammation initiation during ischemia, and amplify the inflammatory response through leukocyte infiltration and further activation of the inflammatory pathway during reperfusion, resulting in an imbalance in tissue repair and increased damage. In addition, the inflammatory response is also associated with other pathological aspects: for example, the release of pro-inflammatory cytokines such as IL-8 and TNF-*α* from microglia leads to hypoxia-inducible factor-α (HIF1α)-dependent autophagic cell death (Takeda et al., 2021), microglia-secreted VEGF can be involved in angiogenesis (An et al., 2014), oxidative stress can trigger inflammatory injury via TLR4 (Gill et al., 2010), NLRP3 can be assembled via NF- kb assembles NLRP3 inflammatory vesicles and promotes caspase-1-mediated pyroptosis (Yang et al., 2019). Clinically, the level of inflammation is also an important factor in assessing treatment, and higher levels of inflammation can represent a poor prognosis (the PAIS investigators the PAIS investigators et al., 2009).

### 2.2 Oxidative stress

The brain consumes 20% of the total basal oxygen to maintain normal neurological function (Cobley et al., 2018), whereas oxygen consumption is high during CI/RI, but its level of antioxidant activity is relatively low, and oxidative stress is prone to occur on this basis. Oxidative stress refers to the disturbance of the balance between the oxidative system and the antioxidant system in the body. Excessive pro-oxidation leads to the destruction of intracellular biomolecules, such as sugars, lipids, proteins, nucleic acids, etc., and then leads to the damage of organs, tissues, and functions, which in turn leads to the further accumulation of oxidative products, resulting in the formation of a vicious circle (Wang et al., 2022b).

#### 2.2.1 Ischemic stage

Excessive production of reactive oxygen species (ROS) and a relative decrease in antioxidant capacity are usually the mechanisms that upset this balance (Orellana-Urzúa et al., 2023), ROS is the core of the oxidation system, which consists of a group of highly reactive ions and molecules produced from molecular oxygen (O_2_), including hydroxyl radicals (•OH), superoxide anions (O_2_•^−^), and non-radical molecules such as hydrogen peroxide (H_2_O_2_) (Galadari et al., 2017). The antioxidant system mainly consists of endogenous antioxidant enzymes such as superoxide dismutase (SOD), catalase (CAT), and glutathione peroxidase (GPX), and the increase in SOD activity counteracts oxidative stress in CI/RI (Zhao et al., 2023b). Under physiological conditions, ROS can be neutralized by SOD to maintain a balance between antioxidant and pro oxidant effects (Olmez and Ozyurt, 2012). Low concentrations of ROS can maintain basic metabolism and redox stability in tissues and cells, which is beneficial for the body. After cerebral ischemia occurs, the hypoxic environment hinders the mitochondrial respiratory chain, leading to ATP production tending toward the anaerobic glycolysis pathway. Glycolysis leads to the accumulation of lactic acid and other acidosis, and the increase in H^+^ converts •O _2_^−^ to H _2_ O _2_ or to more reactive hydroxyl radicals (•OH) (Shirley et al., 2014), while the production of ROS from NADPH increases (Granger and Kvietys, 2015).

#### 2.2.2 Reperfusion stage

During the reperfusion phase, a large amount of ROS begins to flood in and is further generated. The oxygen availability after ischemia resets the mitochondrial respiratory chain, leading to a large increase in superoxide radicals from Complex I sources (O _2_^−^), and a large influx of Ca^2+^ into the mitochondrial permeability transition pore (MPTP) opens up the mitochondria causing swelling and damage ultimately leading to the cessation of ATP synthase and the generation of even more ROS; At the same time, NOX (nicotinamide adenine dinucleotide phosphate oxidase) on the surface of the cell utilizes oxygen as the ultimate electron acceptor via NADPH to immediately produce O_2_^−^, which is another pathway for the massive production of ROS during the reperfusion phase (Chen and Hsieh, 2020). Overall, under physiological conditions, there is a balance between promoting oxidation and antioxidation in the body. CI/RI disrupts this balance, ischemia leads to the initial accumulation of ROS, and reperfusion further exacerbates ROS. Due to the high oxygen/nitrogen demand and metabolism of brain tissue, abundant redox active metals (such as iron and copper), and high levels of oxidizable polyunsaturated fats, its antioxidant capacity is weak and susceptible to ROS damage (Lee et al., 2016). Excess ROS can ultimately cause irreversible damage to brain tissue through lipid peroxidation, damage to mitochondria, and the blood–brain barrier (Figure 1). In addition, ROS not only participates in inflammatory reactions through the mechanisms described in section 2.1, but also mediates autophagy through mitochondria, thereby reducing oxidative stress damage (Filomeni, 2024). It has also been pointed out that ROS can participate in vascular regeneration by regulating HIF-1α and VEGF (Xia et al., 2007).

### 2.3 Mitochondrial dysfunction

Mitochondrial damage is an early initial event in CI/RI (Xu and Hu, 2025), which is widely involved in oxidative stress and inflammatory reaction as the core driving force, and also runs through the whole disease process (Figure 1). Structurally, mitochondria are ovoid or rod-shaped organelles with a double-membrane structure consisting of four distinct compartments: the outer membrane (OMM), the intermembrane space, the inner membrane (IMM), and the matrix. The OMM is the dominant material diffusion barrier and mitochondrial signaling, and contains water channels that allow molecules smaller than 5,000 daltons to diffuse freely into the intermembrane space. The intermembrane space contains proteins (such as cytochrome c), which play a major role in mitochondrial energy and apoptosis. The IMM is the site of the electron transport chain and oxidative phosphorylation, and its permeability is more limited. The inner membrane contains a variety of ion channels and transporters as well as mitochondrial enzyme systems (Liu et al., 2018; Wang et al., 2024a). Functionally, mitochondria are important organelles involved in energy metabolism and calcium homeostasis (Guo et al., 2023a). They are equally involved in immune response, cellular respiration, apoptosis, senescence, and metabolism (Zhou et al., 2021), and are relevant to neuronal survival.

#### 2.3.1 Ischemic stage

Under physiological conditions, the electron transport chain (ETC) can transfer electrons to the final acceptor O_2_, and use proton dynamics (PMF) composed of charge gradient (*Δ*Ψ m) and chemical gradient (Δ pH), and ATP synthase to generate ATP for tissue energy (Berry et al., 2018). After ischemia, the supply of glucose and oxygen is blocked, the disturbance of tricarboxylic acid cycle and the production of ATP are significantly reduced, and the activity and energy supply of ionic pumps such as sodium-potassium pumps on the surface of the cell membrane (Na^+^ − k^+^ -ATPase) as well as Ca^2+^ pumps in the inner mitochondrial membrane are consequently impaired, which results in a large amount of Na^+^ influx, while a dysfunction of endoplasmic reticulum calcium pumps affects Ca^2+^ reuptake, the Na^+^/Ca ^2+^influx caused by mitochondrial dysfunction triggers cellular oedema and swelling at this stage (Wu et al., 2018). Under hypoxia, energy production tends to anaerobic glycolysis, leading to a build-up of lactic acid, which makes the cell pH decline and acidification (Ten and Galkin, 2019) (Figure 1) and hypoxia stops the transmission of electron transport chain (ETC), oxidative phosphorylation is not able to take place, and electrons enter the respiratory chain at a rate greater than the rate of transfer resulting in electron stalling, while the inner mitochondrial membrane potential (ΔΨ m) changes under the condition of ion disorder, so the electron leakage at complexes I and III will increase to generate more o ₂^−^, but at this time, the scavenging effect of low activity SOD on ROS in the ischemic state will decline, and t and the above links finally cause an initial increase in ROS generation (Fang et al., 2025; Hu et al., 2015). Meanwhile, at this stage, ETC oxidative phosphorylation (OXPHOS) is converted to an overactive dephosphorylation state, which creates conditions and basis for ROS burst after reperfusion.

#### 2.3.2 Reperfusion stage

After reperfusion of blood flow, the restoration of O _2_ initiates electron transfer, proton pumping and ATP synthesis, however, in the hyperactive (dephosphorylated) state of the aforementioned ischemic phase, OxPhos hyperpolarizes ΔΨm leading to exponential growth of ROS generation at complexes I and/or III, with a burst of ROS acting as a signal for triggering apoptosis on the one hand, and on the other hand the damage caused by ROS induces mitochondrial dysfunction, which leads to the reduction of electron transport dynamics and ΔΨm levels, further making energy failure (Sanderson et al., 2013). In addition, bursts of ROS and excess accumulation of Ca^2+^ lead to the opening of mitochondrial permeability transition pores (MPTPs) (Halestrap and Richardson, 2015) (Figure 1). The opening of MPTP allows various solutes and water to be released into the mitochondrial matrix, and the inward flow induces rupture and swelling of the inner and outer mitochondrial membranes, leading to necrotic cell death (Wu et al., 2021). Meanwhile, the opening of MPTPs will release two pro-apoptotic factors, mitochondrial cytochrome c (cyt c) and apoptosis-inducing factor (AIF), into the cytoplasm. Upon entering the cytoplasm, AIF interacts with endonuclease G, resulting in DNA breaks (Kumar Saini and Singh, 2024). And Cyt c binds to Apoptosis Protease Activating Factor-1 (Apaf-1), causing Caspase-9 to become more active, which in turn enhances apoptosis (Yang et al., 2018). In addition, MPT can switch from a low conductance state to a high conductance state, and prolonged high conductance will lead to mitochondrial collapse (Dawson and Dawson, 2017).

In general, mitochondrial injury causes impaired energy metabolism and early damage during the ischemic phase, and irreversible damage can continue through ROS burst and structural changes in the reperfusion phase. Since mitochondria are highly dynamic organelles, they change their morphology in response to the many stimuli in CI/RI, which is manifested by the first division driven by cytoplasmic dynamics related protein 1 (Drp1) and the subsequent fusion mediated by mitochondrial fusion protein 1 (mfn1), mitochondrial fusion protein 2 (Mfn2), and optic atrophy protein 1 (OPA1) to form new functional mitochondria, while excessive Division will aggravate CI/RI (Huang et al., 2023a) (Figure 1). Nonfunctional mitochondria can be cleared through the PINK1-Parkin dependent ubiquitination pathway or activated mitochondrial autophagy receptors mediated selective mitochondrial autophagy, thereby reducing ROS production (Shen et al., 2021). Studies have been conducted to attenuate CI/RI in the rat by promoting mitochondrial autophagy and suppressing mitochondrial-mediated apoptosis (Yang et al., 2022).

### 2.4 Programmed cell death in CI/RI

Cell death is essential for the survival and adaptation of multicellular organisms, and under normal physiological conditions, rigorous regulatory mechanisms in the body regulate cell death to maintain normal organismal development and homeostasis (Newton et al., 2024). After ischemia occurs, cells can follow either non-programmed or programmed cell death pathways, depending on the extent of injury (Datta et al., 2020). Unlike non-programmed cell death caused by external injury, namely cell necrosis, apoptosis, ferroptosis, and pyroptosis have been identified as the main forms of programmed cell death involved in CI/RI (Mao et al., 2022; Shu et al., 2022; Zheng et al., 2023).

In CI/RI, irreversible neuronal necrosis occurs in the ischemic center, and cell apoptosis mainly occurs in the ischemic penumbra, and it is reversible within a few hours after ischemia, which is crucial for CI/RI (Hu et al., 2017). Morphologically, apoptosis is characterized by cytoskeletal collapse, nuclear and cytoplasmic condensation, and apoptotic vesicle formation (Zhang et al., 2023). Mechanistically, the apoptotic process is dependent on caspases (Qu et al., 2023), and apoptotic signals can be transduced via mitochondria-mediated endogenous pathways and death receptor-mediated exogenous pathways (Zhang et al., 2022). The status of mitochondria is closely related to the endogenous apoptotic pathway (She et al., 2023). The Bcl-2 family, located upstream of the mitochondria, and the caspase family, located downstream, together play a role in the regulation of apoptosis (Li et al., 2022a). The Bcl-2 family includes pro-apoptotic proteins (such as Bax and Bak) as well as anti-apoptotic proteins (such as Bcl-2 and Bcl-xl), and the proportionality between these two types of proteins is an important determinant of the degree of apoptotic cell death (Valdés et al., 2008); caspases belong to the conserved family of cysteine proteases and can be categorized as either initiator caspases (caspase-8 and caspase-9) or executioner caspases (caspase-3, caspase-6, and caspase-7) according to their mechanism of action in apoptosis (Sahoo et al., 2023). The mitochondrial damage in CI/RI results in a burst of ROS and calcium overload synergized with pro-apoptotic Bax causing the mitochondrial membrane permeability transition pore to open, followed by the release of Cyt C and activation of caspase-9 as described in 2.3 above, which in turn activates caspase-3, ultimately leading to apoptotic cell death (Xu et al., 2021a) (Figure 1). The plasma membrane-located death receptors in the exogenous pathway are tumor necrosis factor family receptors (TNFRs), which bind to tumor necrosis factor family (TNF) such as TNF, Fas ligand (FasL), and TNF-associated apoptosis-inducing ligand (TRAIL), and together with the Fas-associated death structural domain (FADD), they form a death-signaling complex that activates the proteases caspase-8 and later caspase-3, which then induces initiation of apoptosis via protein hydrolysis (Zhao et al., 2023a). In addition, endoplasmic reticulum stress becomes a pro-apoptotic factor after CI/RI, which can also activate caspase family members and initiate apoptosis (Yang et al., 2025a).

Ferroptosis is an iron-dependent programmed cell death distinct from apoptosis and necrosis and is characterized by cell death due to excessive accumulation of lipid peroxides (Li et al., 2022b). Ferroptosis is an important driver of ischemic injury (Stockwell et al., 2017). Intracellular iron exists in the form of divalent iron ions (Fe^2+^) and trivalent iron ions (Fe ^3+^), and iron is widely involved in redox activities in the body as a cofactor for enzymes and catalytic metabolism. Under the condition of Fe ^2+^ enrichment, lipid peroxides can be reduced by Fe ^2+^ to generate highly active alkoxy groups, which can directly destroy adjacent polyunsaturated fatty acids (PUFAs), causing a large amount of lipid related ROS accumulation, leading to Ferroptosis of cells (Yang et al., 2025b). Excessive production of ROS in CI/ RI can cause iron metabolism disorders and increase the sensitivity of the membrane to oxidative stress, leading to membrane lipid peroxidation (LPO) and aggravating ferroptosis (He et al., 2024b). Although the mechanism of ferroptosis in CI/RI is still under study, its biochemical mechanism can be summarized as the formation of iron-dependent lipid reactive oxygen species (L-ROS) and the consumption of glutathione (GSH) or the inactivation of lipid repair enzyme GSH peroxidase 4 (GPX4) (Wang et al., 2021) (Figure 1). It can be seen that ferroptosis in CI/RI is closely related to lipid peroxidation and the antioxidant system, and is closely intertwined with oxidative stress.

Pyroptosis is a novel mode of cell death, defined as highly specific inflammatory programmed cell death, which is mediated by caspase-1 and characterized by the release of cellular contents and pro-inflammatory factors (Li et al., 2023a). The activated inflammasomes such as NLRP3 in CI/RI contribute to the activation of Caspase1, thereby cleaving the precursors of IL-1*β* and IL-18 into mature forms (cl. IL-1β and cl. IL-18), and Caspase-1 can cleave Gasdermin D (GSDMD) to form GSDMD-NT, leading to the formation of pores in the plasma membrane, resulting in cell rupture and the release of inflammatory cell contents, including mature IL-1β and IL-18, which leads to pyroptosis and aggravates the inflammatory response (Luo et al., 2022).

In summary, programmed cell death is extensively involved in all phases of CI/RI temporally, and spatially different patterns of cell death occur at different locations at the injury, which are involved as endpoints in the cascade of various pathological processes (Figure 1).

## 3 Key triggering pathways and their crosstalk in CI/RI

The formation of the macroscopic pathological cascade network in CI/RI is closely related to the regulation of microscopic signaling pathways, which play a crucial role in triggering and amplifying damage in CI/RI. These pathways include TLR/NF-*κ* B, Bcl-2/caspase-9, PINK-Parkin, HIF-1*α* α/VEGF, PI3K/Akt, and Nrf2 mentioned in the previous Section 2, which can promote neuroprotection by regulating these pathways (Bangar et al., 2024; Chen et al., 2020; Lu et al., 2018). The TLR/NF-κ B signaling pathway mainly regulates inflammatory response in CI/RI, which mainly consists of TLRs upstream that recognize pathogen-associated molecular patterns (PAMPs) and DAMPs, MyD88 in the middle, and NF-κ B downstream. There are nine TRLs shared between humans and mice, namely TRL1 ~ TRL9; NF-κB mainly exists in cells in the form of dimers, which are mainly composed of P50 (NF-κB1), p52 (NF-κB2), RelA (p65), Rel B, and c-Rel (Li et al., 2022d). After ischemia, TLR4 can be activated by factors such as HMGB1 (one of the DAMPs mentioned earlier), TNF-α, and IL-1β. Subsequently, it binds to MyD88 to activate IRAK-1 and IRAK-2, members of the IL-1R-associated kinase family, and then IRKs are separated from MyD88 and bind to tnfr-related factor 6 (TRAF6) (Wang et al., 2015). TRAF6, as an E3 ubiquitin ligase, catalyzes the ubiquitination of TAK1 (TGF-β-activated kinase 1), which then phosphorylates IKK-β in the IKK complex, leading to the degradation of i-κB; This allows the NF-κB dimer to dissociate and become activated, ultimately entering the nucleus to upregulate pro-inflammatory factors and gene expression, thereby exacerbating brain injury (Li et al., 2022e; Li et al., 2024). In terms of oxidative stress, Nrf2, as a member of transcription factors, enhances the body’s antioxidant capacity by regulating the expression of antioxidant enzymes. Abnormalities in this pathway can lead to amplified oxidative stress (Esteras and Abramov, 2022). Under physiological conditions, Nrf2 binds to Keap1 located in the cytoplasm to form a dimer, but after oxidative stress occurs, Keap1 undergoes conformational changes due to electrophilic or oxidative signal modification, thereby releasing Nrf2 into the cell nucleus (Liu et al., 2022b). Nrf2 enters the cell nucleus and forms heterodimers with sMaf proteins, which bind to antioxidant response elements (AREs) and initiate gene transcription, then upregulate the levels of antioxidant enzymes (Briones-Valdivieso et al., 2024; Lan et al., 2024). The PINK-Parkin pathway, which is closely related to mitochondrial dynamics, initiates mitochondrial autophagy through a ubiquitination cascade to control mitochondrial quality (Tanaka, 2020) (Figure 1). For apoptosis, PI3K/Akt is a key pathway, with the main members are phosphatidylinositol 3-kinase (PI3K) and protein kinase B (Akt); The initiation of this pathway begins with the activation of growth factor receptors, which recruit PI3K to the membrane and promote its activation, while Fully activated PI3K converts 4,5-diphosphate phosphatidylinositol (PIP2) into PIP3, and then PIP3 activates Akt through 3-phosphinosidine-dependent protein kinase 1 (PDK1) to regulate a series of downstream pathological processes, such as reducing caspase-mediated apoptosis by downregulating Bax, as mentioned in Section 2.4 (Huang et al., 2022; Zheng et al., 2024). In addition, Akt activation can also be mediated by mTORC2, which increases the overall level of Akt activity during the disease process (Huang and Manning, 2009). In terms of vascular regeneration, the key regulatory nodes revolve around VEGF, and upregulation of the HIF-1*α*/VEGF pathway can promote neural recovery in CI/RI (Li et al., 2022c).

**Figure 2:**
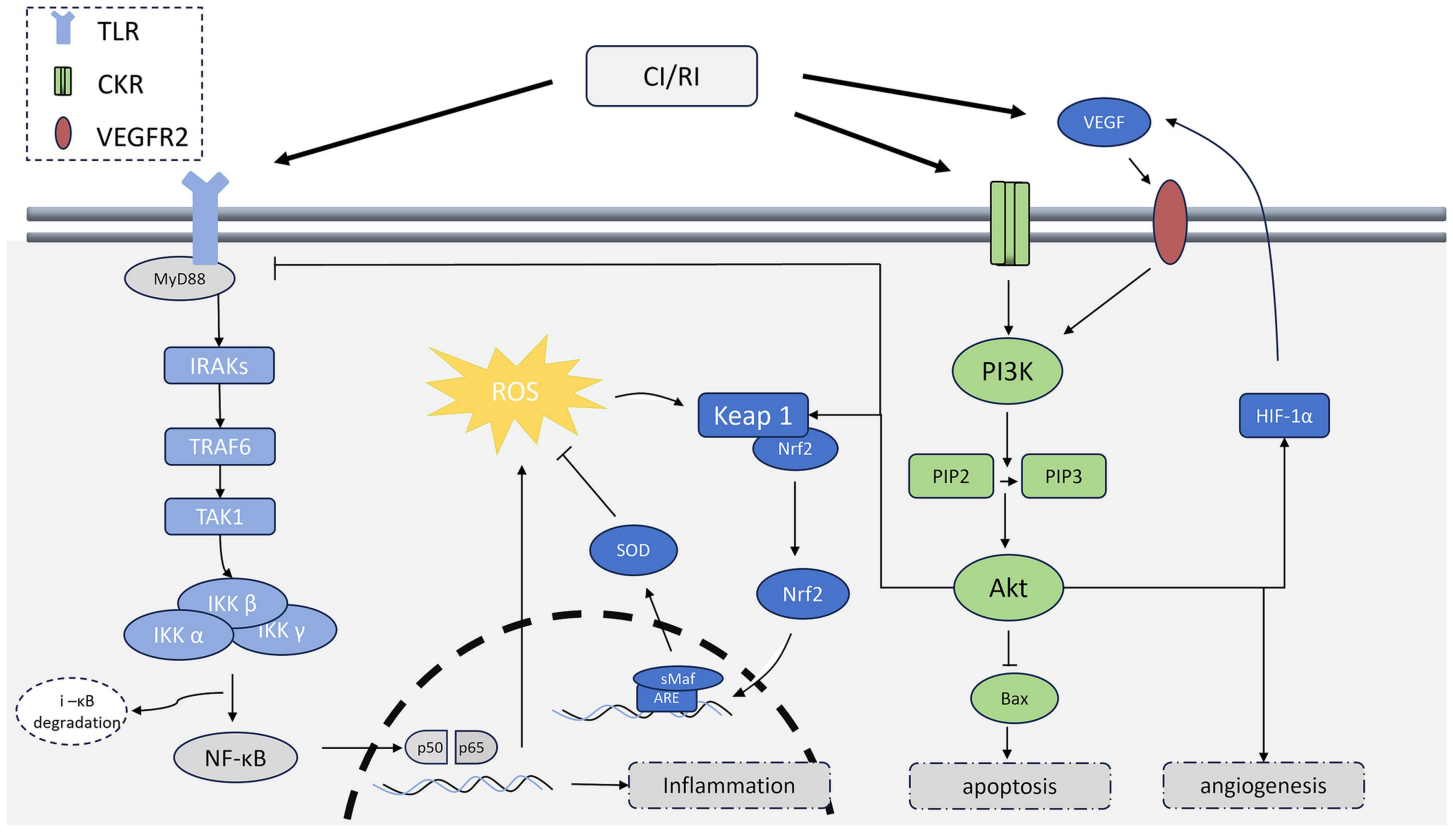
This figure illustrates the crosstalk and interplay between key signaling pathways regulating CI/RI, which collectively regulate downstream pathological processes.

It is worth noting that the above-mentioned key pathways not only regulate the pathological processes in CI/RI separately, but also form complex crosstalk through key nodes, thereby jointly regulating CI/RI (Figure 2). For example, the Nrf2 pathway is associated with the TLR/NF-κB pathway through ROS: Activation of NF-κB increases ROS levels, and based on this situation, the Nrf2 pathway downregulates ROS to achieve joint regulation of inflammatory responses and oxidative stress. Nrf2 can also be linked to activated Akt via Keap1, which manifests as activation of the PI3K/Akt pathway, promoting the dissociation of Nrf2 and Keap1, and comprehensively exerting neuroprotective effects (Liu et al., 2025). The PI3K/Akt pathway can also inhibit TLR4 expression to establish a connection with the NF-κB pathway; Studies have shown that upregulating PI3K/Akt will block the NF-κB pathway, thereby exerting anti-inflammatory effects (Li et al., 2023b; Zhu et al., 2018). The activation of the PI3K/Akt pathway can also promote an increase in HIF-1α level, which upregulates VEGF expression, and VEGF then activates the PI3K/Akt pathway through the VEGFR2 receptor on the cell membrane, thus amplifying the protective effect on neurovascular units (Karar and Maity, 2011; Wang et al., 2022a). In contrast, some pathways could exacerbate brain damage by negative crosstalk. For example, the Wnt/*β* signaling pathway promotes the degradation of i-κB, thereby upregulating the NF-κB pathway and aggravating the inflammatory response (Zhang et al., 2024a). It is apparent that crosstalk is essential for maintaining the delicate balance between CI/RI and various pathological processes, but it also increases the difficulty of precision treatment to a considerable extent.

## 4 Targeted therapies for pathologic processes in CI/RI

### 4.1 Targeted inflammatory response and its mediated pyroptosis

As described in 2.1, the inflammatory response causes injury in CI/RI by driving a storm of pro-inflammatory factors through the release of DAMPs, and key intervention nodes include upstream targets: TLR4, NLRP3, midstream signaling under the NF-kB pathway, and downstream effector molecules: IL-1β, TNF-α, and ICAM-1, etc. ApTOLL, a DNA-binding aptamer, is an antagonist of TLR4 that reduces its activity and impedes its binding to DAMPs, thereby blocking the TLR4/MyD88/NF-kB pathway to attenuate the inflammatory cascade, and its efficacy has been demonstrated in preclinical phases (in rat cerebral ischemia/reperfusion animal models) (Fernández et al., 2018), and it has demonstrated a superior safety profile in human phase I clinical trials (FIH I study) (Hernández-Jiménez et al., 2022). A double-blind, randomized, multicenter, placebo-controlled study in combination with EVT is currently advancing this targeted agent into Phase Ib/IIa clinical studies (APRIL study) (Hernández-Jiménez et al., 2023). Restatorvid (TAK-242), another TLR4-targeted inhibitor, significantly reduced the infarct size and edema degree after cerebral ischemia and hypoxia in rats in an *in vivo* study (Jiang et al., 2020). TJ-M2010-5 (TJ-5), a novel CNS drug candidate with good blood–brain barrier permeability, is a specific inhibitor of MyD88, which also blocks signaling in the TLR4/MyD88/NF-kB pathway, and a study has shown that this targeted inhibitor significantly suppressed the excessive inflammatory response in a rat model of cerebral ischemia–reperfusion injury and reduced the rat cerebral infarction area (Li et al., 2022f).

As mentioned in 2.4, NLRP3 inflammasome activation is a key motivator of pyroptosis in CI/RI. MCC950 is a specific inhibitor of NLRP3, and this targeted drug significantly slowed down the pyroptosis in the ischemic penumbra of mouse brain tissue in the preclinical stage (*in vivo* experiment) and simultaneously inhibited the reverse-promoting effect of pyroptosis on inflammation (Bellut et al., 2023; Bellut et al., 2021). In recent years, an in vivo experimental study has used a novel functional nanoparticle delivery system (MM/ANPs) to deliver Apelin-13 to the damaged site through the blood–brain barrier and inhibit NLRP3 inflammasome assembly by enhancing sirtuin 3 (SIRT3) activity, providing a new strategy for targeted inhibition of inflammatory response and pyroptosis (Ma et al., 2025).

In addition, it has been described in 2.1 that microglia are the first to polarize and transform into different M1 phenotypes (pro-inflammatory) and M2 phenotypes (anti-inflammatory) in the inflammatory response. Minocycline has significant anti-inflammatory effects on the nervous system by targeting microglia thereby inhibiting their activity and promoting the transition of microglia from M1 to M2 (Lu et al., 2021). The safety of therapeutic doses of minocycline has been demonstrated, and a multicenter, prospective, randomized, open-label, blinded endpoint clinical study was currently designed to investigate its efficacy and safety in combination with mechanical thrombus retrieval (MT) in patients with acute stroke (MIST-A study) (Zhang et al., 2024b). A study reported a highly efficient neutrophil hijacking nanoplatform (called APTS), which uses the natural transport properties of neutrophils to cross the BBB to accurately target telomerase repeats (A151) to microglia to make it transition to the M2 phenotype, significantly reducing neuroinflammation and infarct size in mice (Yin et al., 2024).

As stated in section 2.1, the infiltration and adhesion of leukocytes are important factors in amplifying inflammatory responses, which is critically facilitated by adhesion molecules such as p-selectin, e-selectin, and ICAM-1. Enlimomab, an ICAM-1-specific antibody, reduced leukocyte adhesion and infarct size in mice in preclinical experiments, but an early multicenter, randomized, placebo-controlled clinical trial of its treatment of acute stroke was associated with serious safety concerns, and the targeted agent is currently in an improvement phase (Enlimomab Acute Stroke Trial Investigators, 2001). Due to the failure of anti-ICAM-1 in human trials, a blinded placebo-controlled trial of non-human primate stroke significantly improved the neurological function of treated animals and significantly reduced the infarct size by using huep5c7 (human monoclonal antibody against e-selectin and p-selectin), which provided a new possibility for the treatment strategy of anti-inflammatory response by blocking adhesion molecules and leukocyte infiltration (Mocco et al., 2002).

Moreover, the efficacy of anti-inflammatory strategies that directly target pro-inflammatory factors has been validated, with Canakinumab targeting IL-1β in a randomized, double-blind trial, which reduced inflammation levels and stroke incidence in the study population (Everett et al., 2020). In conclusion, targeting the inflammatory response can start from multiple dimensions, from inhibiting upstream pathways (such as TLR4 and NLRP3) to regulating cellular phenotypes (such as microglial polarization), and then to innovative delivery technologies (such as nanoparticles) (Table 1).

**Table 1:** Targeted drugs and their effects on inflammation in CI/RI.

Drugs	Effect	Target	Mechanism	Clinical phase	Drug limitations
ApTOLL (Fernández et al., 2018; Hernández-Jiménez et al., 2023)	Anti-inflammation	TLR4	Antagonizing TLR4 blocks the inflammatory cascade signal transduction.	Phase II	Limited BBB penetration
Resatorvid (TAK-242) (Jiang et al., 2020)	Anti-inflammation	TLR4		Preclinical stage	Low bioavailability
TJ-M2010-5(TJ-5) (Li et al., 2022f)	Anti-inflammation	MyD88		Preclinical stage	Off-target effects
Minocycline (Lu et al., 2021; Zhang et al., 2024b)	Anti-inflammation	microglia	Promote the transformation of microglial phenotype from M1 to M2	Phase II	Adverse reactions
APTS (Yin et al., 2024)	Anti-inflammation	microglia		Preclinical stage	Challenges in nanocarrier loading efficiency
Enlimomab (Enlimomab Acute Stroke Trial Investigators, 2001)	Anti-inflammation	ICAM-1	Anti-leukocyte adhesion	Preclinical stage (under improvement)	Drugs safety requires further research
HuEP5C7 (Mocco et al., 2002)	Anti-inflammation	e-selectin/p-selectin	Anti-leukocyte adhesion	Preclinical stage	Insufficient research for Drug-effectiveness
Canakinumab (Everett et al., 2020)	Anti-inflammation	IL-1β	Reduce proinflammatory factor levels	Phase II	Infection risk increases
MCC950 (Bellut et al., 2023; Bellut et al., 2021)	Anti-inflammation, Anti-pyroptosis	NLRP3	Blocking the activation of NLRP3	Preclinical stage	Hepatic toxicity
Apelin-13 (Ma et al., 2025)	Anti-inflammation, Anti-pyroptosis	NLRP3	Inhibition of the assembly of NLRP3	Preclinical stage	Drug safety assessment is insufficient

### 4.2 Targeting oxidative stress and its mediated ferroptosis

As described in 2.2 and 2.4, both oxidative stress and ferroptosis are closely related to the weakening of the antioxidant system and the enhancement of the oxidative system *in vivo*. The key interventions include enhancing the activity of antioxidant enzymes such as SOD and GPX4 and scavenging or inhibiting the excessive generation of ROS. Edaravone (MCI-186), which targets the scavenging of oxygen free radicals to reduce ROS (Ma et al., 2022), has demonstrated safety and efficacy in stroke patients, and its efficacy and therapeutic time window were further investigated in a multicenter, prospective, randomized, and open-label study of combined thrombolytic therapy (the YAMATO study) (Aoki et al., 2017), and this drug is currently in the listing stage. Melatonin can also scavenge free radicals to reduce ROS levels (Chitimus et al., 2020). A comprehensive study of *in vivo* experiments and randomized controlled clinical trials showed that melatonin reduced the infarct size of rat brain tissue by anti-oxidative stress, and the level of antioxidant damage in patients receiving melatonin treatment was increased (Zhao et al., 2018).

As mentioned in 2.4, free Fe^2+^ enrichment can lead to pro-oxidation and ferroptosis. Deferoxamine (DFO) is an iron chelating drug that targets free pro-oxidized iron, blocking and attenuating iron-dependent ROS production and iron death, and it significantly attenuated CI/RI injury in animal models in preclinical studies (Van Der Loo et al., 2020), and in recent years, a combination of intravenous recombinant tissue-type plasminogen activator (tPA) has been shown to be effective against stroke patients efficacy of a multicenter, randomized, double-blind, placebo-controlled, dose-discovery phase II clinical trial validated the favorable safety profile of this targeted drug (Millán et al., 2021). Ferrostatin-1 is a specific ferroptosis inhibitor (Chen et al., 2025), which can target the removal of lipid ROS (L-ROS) to block ferroptosis and enhance the body’s antioxidant capacity, but it has the limitation of circulating inactivation. In an *in vivo* experiment, BBB-targeted lipid nanoparticles were used to encapsulate Fer1 to prepare T-LNPs-Fer, which can cross the BBB to enhance the utilization of Ferrostatin-1, maintain the homeostasis of GPX4 in neuronal cells after cerebral ischemia, and significantly inhibit ferroptosis oxidative stress in rats (Shi et al., 2024a).

In addition, the enhancement of the antioxidant system was related to nuclear factor erythroid 2-related factor 2 (NRF2). Sulforaphane is a targeted agonist of NRF2, which can enhance the activities of SOD, heme oxygenase 1 (HO-1) and GPX4 to resist oxidative stress and ferroptosis. An *in vivo* study using this targeted drug to upregulate NRF2 reduced CI/RI in rats (Fan et al., 2024). In short, the treatment of oxidative stress and ferroptosis can start from controlling the body’s anti-oxidation and pro-oxidation balance (Table 2).

**Table 2:** Targeted drugs and their effects on oxidative stress in CI/RI.

Drugs	Effect	Target	Mechanism	Clinical phase	Drug limitations
Edaravone (MCI-186) (Ma et al., 2022; Aoki et al., 2017)	Anti-oxidative stress	ROS	Scavenging free radicals	Phase IV	Drug nephrotoxicity, Limited therapeutic window
Melatonin (Chitimus et al., 2020; Zhao et al., 2018)	Anti-oxidative stress	ROS	Scavenging free radicals	Phase II	Hepatic toxicity
Sulforaphane (Fan et al., 2024)	Anti-oxidative stress	NRf2	Enhance antioxidant enzyme activity	Preclinical stage	Unstable chemical properties
Deferoxamine (DFO) (Van Der Loo et al., 2020; Millán et al., 2021)	Anti-oxidative stress, Anti-ferroptosis	Fe^3+^	Blocking the production of L-ROS	Phase II	Limited BBB penetration
Ferrostatin-1 (Chen et al., 2020; Shi et al., 2024a)	Anti-oxidative stress, Anti-ferroptosis	L-ROS	Blocking the production of L-ROS	Preclinical stage	Drugs safety requires further research

### 4.3 Targeting mitochondrial dysfunction and its mediated apoptosis

As described in 2.3, mitochondrial damage is an important source of ROS bursts in CI/RI and drives endogenous apoptosis in the ischemic penumbra, which involves ETC electron leakage, MPTP over-opening, apoptotic signaling release, and imbalance in mitochondrial dynamics. Mitoquinol (MitoQ) is an antioxidant targeting mitochondria, which is able to highly accumulate in the inner mitochondrial membrane and reduce ROS production, thus attenuating the cascade of pathological links, and this targeted drug has shown significant neuroprotection in rat models by inhibiting mitochondrial damage in preclinical studies (Ibrahim et al., 2023). While the potential of this targeted agent for clinical applications in CI/RI remains unknown, a double-blind, placebo-controlled study in the field of Parkinson’s disease emphasized its safety (Snow et al., 2010) (Table 3).

**Table 3:** Targeted drugs and their effects on mitochondrial dysfunction in CI/RI.

Drugs	Effect	Target	Mechanism	Clinical phase	Drug limitations
Mitoquinol (MitoQ) (Ibrahim et al., 2023; Snow et al., 2010)	Anti-mitochondrial dysfunction	mitochondria	Aggregation in the IMM reduces ROS production	Phase I	Adverse reactions
P110 (Hao et al., 2023; Liu et al., 2022c)	Anti-mitochondrial dysfunction	Drp1	Reduce excessive mitochondrial fission	Preclinical stage	Insufficient research for Drug-effectiveness and safety
Ginsenoside compound K(CK) (Huang et al., 2023b)	Anti-mitochondrial dysfunction	Mfn2	Promote mitochondrial fusion	Preclinical stage	Low solubility and bioavailability
S89 (Guo et al., 2023b)	Anti-mitochondrial dysfunction	Mfn1		Preclinical stage	Insufficient research for Drug-effectiveness and safety about CI/RI
MTX115325 (Fang et al., 2023)	Anti-mitochondrial dysfunction	Parkin	Enhance mitophagy	Preclinical stage	Hepatic toxicity, Off-target effects
Cyclosporine A (CsA) (Chen et al., 2021a; Nighoghossian et al., 2015; Liu et al., 2022a)	Anti-mitochondrial dysfunction, Anti-apoptosis	mPTP	Inhibit the excessive opening of mPTP	Phase II	Immunosuppressive side effects
Icariin (Zhou et al., 2024b)	Anti-mitochondrial dysfunction, Anti-apoptosis	mPTP	Inhibit the excessive opening of mPTP	Preclinical stage	Limited drug delivery efficiency, Low bioavailability
Oriidonin (Ori) (Li et al., 2023c)	Anti-apoptosis	caspase-9	Blocking the signal transduction of apoptosis	Preclinical stage	Insufficient targeting accuracy

Cyclosporin A (CsA) binds to cyclophilin D in mitochondria, targets MPTP and inhibits its excessive opening, thereby attenuating apoptosis and mitochondrial damage, and it has shown promising efficacy in in vivo experiments (animal models) (Chen et al., 2021a). A subgroup analysis of a phase II clinical study of combined thrombolytic therapy for stroke suggests that CsA can significantly reduce the growth of infarct volume in patients with successful recanalization, but its efficacy still needs further phase III studies (Nighoghossian et al., 2015). Recent studies have improved BBB integrity and reduced cerebral infarct area in MCAO mice by preparing CsA-loaded HFn nanoparticles (CsA@HFn) to specifically deliver CsA to cerebral ischemic areas, presenting a new hope for specifically targeting MPTP (Liu et al., 2022a). Meanwhile, the discovery of new MPTP-inhibiting drugs is also underway, such as Icariin, which improved cerebral ischemia–reperfusion injury in rats by inhibiting the excessive opening of MPTP (Zhou et al., 2024b).

In terms of mitochondrial dynamics, targeted therapy strategies mainly focus on reducing excessive fission and promoting effective fusion. P110 is a peptide inhibitor that can target the inhibition of short-term protein interaction in the Drp1 group to reduce the excessive division of mitochondria (Hao et al., 2023). A preclinical study improved the targeting efficiency by preparing macrophage-derived exosomes (EXO-Hep) loaded with the targeted drug, which significantly reduced cerebral ischemia–reperfusion injury in rats (Liu et al., 2022c). Mfn2 can mediate mitochondrial fusion repair. An in vivo study pointed out that ginsenoside compound K (CK) can increase the expression of Mfn2, and then play a protective role in rat neurons by regulating mitochondrial dynamics (Huang et al., 2023b). Meanwhile, a new specific agonist, S89, was reported in a study to target Mfn1 to promote mitochondrial fusion, which significantly ameliorated ischemia–reperfusion injury in the mouse heart (Guo et al., 2023b), which may shed light on its application in CI/RI. The targeted therapeutic strategy for mitophagy is generally to enhance autophagy to eliminate damaged mitochondria, and MTX115325, which is an inhibitor of USP30, was able to indirectly target Parkin and thus enhance mitochondrial autophagy, which exerted significant neuroprotective effects in mice (Fang et al., 2023).

For apoptosis, in addition to protecting the mitochondrial state, considering the hierarchical signal transduction, targeting the Caspase cascade in its downstream execution phase is also a therapeutic strategy. In an in vivo experiment, oridonin (Ori) was used to specifically inhibit caspase-9-mediated neuronal apoptosis and reduce reperfusion injury in mice (Li et al., 2023c). In addition, apoptosis is also regulated by upstream complex signaling pathways (PI3K/Akt, AMPK, etc.), and currently targeted therapies for these pathways are mainly focused on the preclinical research stage (Chen et al., 2020; Chen et al., 2021b; Wang et al., 2022c).

## 5 Discussion and prospects

In summary, CI / RI involves a variety of complex pathological processes, which are involved in the process from cerebral ischemia to reperfusion and recovery, and each of them follows a specific pathological cascade mechanism (Figure 1). In the past studies, specifically targeted drugs for the key stages of various pathological links have provided significant effects and progress in the treatment of CI/RI (Table 1). However, it is obvious that the complex pathologic cascades are both temporally and spatially intertwined, and their upstream regulatory pathway is also complex and has crosstalk, which suggests to us that targeted therapeutic strategy still has great research potential and its limitations can be perceived.

First of all, although the blockade of a single target can achieve precise intervention, it cannot block the complex network of pathological processes. For example, edaravone focuses on reducing oxidative stress, but cannot block cytokine cascades and ETC disorders in the inflammatory response. Edaravone dextroborneol (EDB) is a novel neuroprotective agent composed of Edaravone, which can scavenge free radicals, and Dexborneol, which can block the cascade of multiple inflammatory factors, targeting both oxidative stress and inflammatory responses (Wang et al., 2024b). In a randomized, double-blind, comparative phase III clinical trial study, good functional outcome at 90 days was superior to that of the edaravone-alone group in Chinese stroke patients treated with edaravone dextroamphetamine (EDB) within 48 h (Xu et al., 2021b). Traditional Chinese medicine has the characteristics of multi-pathway and multi-target synergy, and its research potential has been reflected in a variety of preclinical studies (Shi et al., 2024b). For example, DHI, composed of *Salvia miltiorrhiza* and *Carthamus tinctorius* in a 3: 1 ratio, can comprehensively alleviate CI/RI in rats by upregulating mitochondrial dynamics-related proteins such as Mfn1, Mfn2, and OPA1 and downregulating apoptosis-related pathway proteins such as Cyt c, Apaf1, Bax, Caspase-3, and Caspase-9 (Du et al., 2023). As well as the traditional Chinese medicine decoction Buyang Huanwu Tang, which formula is mainly composed of Huangqi, with effective chemical components including kaempferol, quercetin, paeoniflorin, astragaloside IV, Hydroxysafflor Yellow A and Glycosides, exerts its effects by upregulating the AKT/TP53 and PINK1/Parkin signaling pathways and downregulating the Nrf2/GPX4 pathway, thereby influencing multiple pathological processes such as inflammation and oxidative stress, and has shown neuroprotective effects in rats (Cao et al., 2024; Jiao et al., 2024; Yin et al., 2023). This evidence suggests to us that rational and effective multi-target combination therapy may be a promising approach to break the multi-pathological cascade.

Secondly, the precise and effective delivery of specifically targeted drugs to their targets is inevitably challenged by the presence of the BBB, such as CsA, which was mentioned in 3.3. Nanocarrier drugs have been identified as an ideal targeted therapeutic strategy for cerebral ischemia, with the ability to efficiently deliver drugs to the damaged brain parenchyma and further to specific cells, such as neurons or glial cells, with an increased chance of drug targeting due to the longer circulation time required for nanodrug delivery (Li et al., 2021). Studies have used tetramethylpyrazine (TMP) nanoparticles to reduce CI/RI in mice (Mu et al., 2023). Another study used macrophage-derived exosomes (Ex) to load curcumin (cur) to form (Ex-cur), which improved the stability of cur in rats and significantly and efficiently played a neuroprotective role in rats (He et al., 2020). It can be seen from these that nanocarriers may be a promising strategy to improve the efficiency of targeted therapy. In addition, emerging cell therapies are also promising targeted treatment strategies. For example, mesenchymal stem cell therapy can migrate to damaged brain tissue through the homing effect and paracrine neuroprotective factors to alleviate CI/RI (Rust et al., 2024).

Additionally, although this review systematically describes the pathological processes of inflammation, oxidative stress, and mitochondrial damage that play important roles in the progression of CI/RI, the overall pathological mechanism of CI/RI is extensive and complex. This paper adopts a relatively macro perspective as its approach for integration and elaboration. Therefore, in the CI/R, in addition to the key cascade points mentioned in this paper, members of relatively secondary response links can also serve as starting points to play a role in the pathological cascade. For example, neutrophils, as one of the recruited leukocytes after the initiation of inflammatory response, can not only aggravate inflammation by secreting pro-inflammatory factors such as IL-1 *β*, TNF - *α*, IL-6, but also provide impetus for the occurrence of oxidative stress by producing ROS and RNS through respiratory burst; Moreover, they exert a protective effect through phagocytosis in the early stages of CI/RI, while in the later stages, they impair vascular remodeling by forming neutrophil extracellular traps (NETs), and this bidirectional effect is also worthy of attention (He et al., 2024a). At the cellular level, similar to the microglia mentioned in this article, astrocytes, which are an important component of the BBB, can also be activated into two different phenotypes, A1 and A2, during the course of the disease. Following ischemic injury, A1 promotes glial scar formation through glial fibrillary acidic protein, thereby impairing axonal growth, and secretes soluble neurotoxins such as Serping1, H2-D1, and Ggta1, which damage neurons and exhibit neurotoxic effects; Type A2, on the other hand, participates in axonal regeneration and BBB repair through platelet-derived growth factor (PDGF) in the later stages of the disease (Cheng et al., 2025). Astrocytes are just one component of the BBB, BBB damage is a key link in the initiation and amplification of CI/RI, and an intact BBB can effectively protect brain tissue from damage caused by various pathological factors (Feng et al., 2024). Recent studies have begun to focus on vascular neural units, which place greater emphasis on overall protection, such as, regulating the Wnt/Beta-Catenin signaling pathway can exert a protective effect on CI/RI at multiple levels, including endothelial cells, neurons, oligodendrocytes, microglia, and glial cells (Mo et al., 2022). The aforementioned researches emphasize the spatiotemporal complexity of CI/RI. In addition to various cytokines and substances entering the bloodstream, different types of intrinsic cells are also an important part of the pathological cascade network, which is why a multidimensional interference method that can take into account the characteristics of different stages of CI/RI and considers the body’s intrinsic structures, such as the BBB, from both a temporal and spatial perspective may be a promising approach in order to cover this complex dynamic process.

## 6 Conclusion

All The pathological mechanism of CI/RI involves a dynamic intertwining of multiple components, with inflammation, oxidative stress, and mitochondrial dysfunction collectively exacerbating brain injury through a complex network of signaling pathways. Existing targeted therapy strategies (such as TLR4 inhibitors, ferroptosis antagonists, and mitophagy regulators) have shown significant efficacy in preclinical studies, but single-target intervention is difficult to cope with the spatiotemporal complexity of the pathological network. To overcome this limitation, combination therapy (such as anti-oxidation-anti-inflammatory synergy) and nano-delivery technology can improve the efficiency of intervention. Therefore, it is meaningful to conduct in-depth studies to elucidate the complex pathological links and the crosstalk mechanism among pathways. With the deepening of the mechanism research, the development of precise nano-delivery systems and the multi-target combination therapies may be a promising strategy to ultimately realize the efficient prevention and treatment of CI/RI.
